# A Good Appliance

**Published:** 1899-07

**Authors:** 


### A Good Appliance.

We have for some time been using an instrument devised and made by Dr. F. F. Hoyer, of Owosso, Mich. It is an instrument designed for protecting the mouth and lips while using a corundum disk, polishing disk, diamond disk and fine circular saw. This appliance is used for protecting the teeth while separating them, for dressing them, for finishing fillings, etc.

Almost every one is aware of the fact that in the use of these, unless great care is taken, injury is very likely to be done to the soft tissues. Dr. Hoyer's shield is an entire security against injury in the use of either of the above-mentioned disks. We know of nothing equal to this appliance. It ought to be in the possession of every dentist.

